# Profiling and functional characterization of long noncoding RNAs during human tooth development

**DOI:** 10.1038/s41368-025-00375-7

**Published:** 2025-05-09

**Authors:** Xiuge Gu, Wei Wei, Chuan Wu, Jing Sun, Xiaoshan Wu, Zongshan Shen, Hanzhang Zhou, Chunmei Zhang, Jinsong Wang, Lei Hu, Suwen Chen, Yuanyuan Zhang, Songlin Wang, Ran Zhang

**Affiliations:** 1 Beijing Key Laboratory of Tooth Regeneration and Function Reconstruction, Beijing Laboratory of Oral Health and Beijing Stomatological Hospital, Department of Biochemistry and Molecular Biology, Capital Medical University School of Basic Medical Sciences, Capital Medical University, Beijing, China; 2 Academician Workstation for Oral-Maxillofacial Regenerative Medicine, Central South University, Changsha, China; 3 Hospital of Stomatology, Guangdong Provincial Key Laboratory of Stomatology, Guanghua School of Stomatology, Sun Yat-sen University, Guangzhou, China; 4 Department of Oral Pathology, Peking University School and Hospital of Stomatology & National Center of Stomatology & National Clinical Research Center for Oral Diseases & National Engineering Research Center of Oral Biomaterials and Digital Medical Devices, Beijing, China; 5 Department of Reproductive Regulation, Beijing Obstetrics and Gynecology Hospital, Beijing Maternal and Child Health Care Hospital, Capital Medical University, Beijing, China; 6 Laboratory of Homeostatic Medicine, School of Medicine, Southern University of Science and Technology, Shenzhen, China

**Keywords:** Developmental biology, Cell biology

## Abstract

The regulatory processes in developmental biology research are significantly influenced by long non-coding RNAs (lncRNAs). However, the dynamics of lncRNA expression during human tooth development remain poorly understood. In this research, we examined the lncRNAs present in the dental epithelium (DE) and dental mesenchyme (DM) at the late bud, cap, and early bell stages of human fetal tooth development through bulk RNA sequencing. Developmental regulators co-expressed with neighboring lncRNAs were significantly enriched in odontogenesis. Specific lncRNAs expressed in the DE and DM, such as *PANCR*, *MIR205HG*, *DLX6-AS1*, and *DNM3OS*, were identified through a combination of bulk RNA sequencing and single-cell analysis. Further subcluster analysis revealed lncRNAs specifically expressed in important regions of the tooth germ, such as the inner enamel epithelium and coronal dental papilla (CDP). Functionally, we demonstrated that CDP-specific *DLX6-AS1* enhanced odontoblastic differentiation in human tooth germ mesenchymal cells and dental pulp stem cells. These findings suggest that lncRNAs could serve as valuable cell markers for tooth development and potential therapeutic targets for tooth regeneration.

## Introduction

Tooth development is a complex biological process that involves interactions between dental epithelium (DE) and underlying dental mesenchyme (DM), along with precise regulation of gene expressions.^1–4^ This process in humans begins around 7–8 weeks of gestation and progresses through the bud, cap, and bell stages. During these stages, a variety of signaling molecules, transcription factors, and regulatory elements coordinate to ensure proper tooth development. Studying the gene expression dynamics during tooth development is crucial for comprehending the biological processes involved, recognizing dental developmental disorders, and devising tooth regeneration strategies.

Long non-coding RNAs (lncRNAs) play a crucial role in regulating gene expression through various mechanisms in developmental biology research. These non-coding RNA molecules, which are over 200 nucleotides long, are involved in transcriptional, epigenetic, and post-transcriptional regulation.^5^ Research on particular categories of lncRNAs in human and mouse embryonic stem cells has revealed their significance in regulating the expression of developmental genes as cells differentiate.^6,7^ Additionally, lncRNAs are essential for the development of various tissues and organs, such as the nervous system, the lung, the heart, and skeletal structures.^8–11^ Recent research has also uncovered the relationship between lncRNAs and tooth development. Certain lncRNAs, like *H19*, *DANCR*, and *MEG3*, have been identified as regulators of the differentiation of dental-derived mesenchymal stem cells, targeting important signaling pathways such as WNT, BMP.^12–16^ Our previous study indicated the involvement of lncRNAs in extracellular matrix establishment during miniature pig deciduous tooth development.^17^ Given the conservation of lncRNAs across species and their location-based functions, it is important to investigate the dynamics of lncRNA expression in humans with location-specific information.

Bulk RNA sequencing (bulk RNA-seq) is widely used to analyze the expression profiles of lncRNAs in tissue development.^18,19^ It enables the detection of low-abundance transcripts, provides a broad overview of gene expression patterns, and is cost-effective for large sample sizes.^20–22^ However, bulk RNA-seq provides average gene expression profiles of tissues, which cannot capture the cellular heterogeneity within complex tissues. Our study performed a comprehensive analysis of lncRNA and messenger RNA (mRNA) expression profiles by isolating DE and underlying DM during various stages of human tooth development using bulk RNA-seq. The integration of single-cell RNA sequencing with bulk RNA-seq has uncovered the involvement of lncRNA in particular cell populations in recent studies.^23,24^ Additionally, our study incorporated previously published single-cell combinatorial-indexing RNA sequencing (sci-RNA-seq) and our recently published spatial transcriptomic datasets of human fetal teeth to investigate lncRNAs specifically expressed in key odontogenic subpopulations, such as inner enamel epithelial (IEE) cells and coronal dental papilla (CDP) cells, with the goal of identifying regulators of enamel and dentin formation for tooth regeneration.

## Results

### Identification of lncRNAs of human tooth germs and investigation of their *cis*-regulatory potential in odontogenesis

**Fig. 1:**
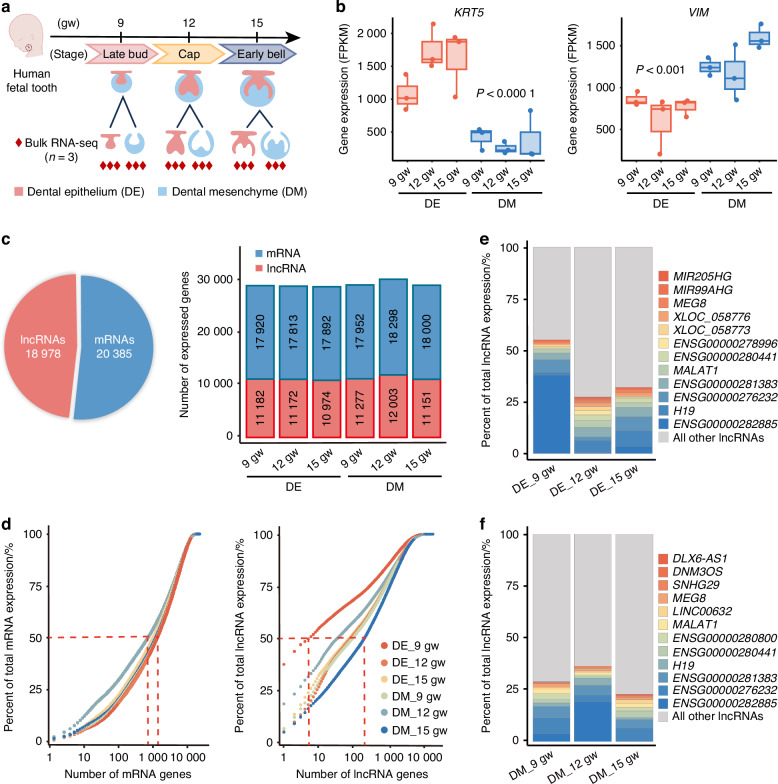
Identification of lncRNAs in developing fetal teeth. **a** Schematic diagram of bulk RNA-seq samples from dental epithelium (DE) and dental mesenchyme (DM) of developing fetal teeth at the late bud, cap, and early bell stages. *n* = 3 per group. gw gestational week. **b** Boxplots showing the expression (FPKM) of *KRT5* and *VIM* in DE and DM across the three developmental stages, through bulk RNA-seq. **c** Pie graph showing the quantity of robustly expressed mRNAs and lncRNAs. The number of genes expressed in DE and DM across the three developmental stages is also shown. **d** Cumulative expression plots comparing the number of mRNAs (left) and lncRNAs (right), that constitute the total sum of gene expression (FPKM), respectively. Percentage of total lncRNA expression (FPKM) accounted for by the union of the top 12 most highly expressed lncRNAs in DE (**e**) and DM (**f**)

Tooth development progresses through several stages, each of which plays a crucial role in the formation of the tooth. These stages are commonly referred to as the bud, cap, and bell stages, and they are part of a continuous process of tooth morphogenesis.^2,25^ The bud stage marks the initiation of tooth development and establishes signaling mechanisms for subsequent morphogenesis. The cap stage is marked by the initial differentiation of DE and DM, while the bell stage is crucial for enamel and dentin formation. During these stages, the DE and DM serve distinct roles characterized by distinct molecular expression patterns.^26^ This study performed bulk RNA-seq on isolated DE and DM from developing fetal teeth at the late bud, cap, and early bell stages (Fig. 1a). The epithelial marker *KERATIN 5* (*KRT5*) showed strong expression in the DE across all three stages, while the DM marker *VIMENTIN* (*VIM*) was predominantly expressed in the DM, confirming successful tissue separation (Fig. 1b). Our analysis identified a total of 18 978 lncRNAs and 20 385 mRNAs, with robust expression of both lncRNAs and mRNAs in the DE and DM throughout all developmental stages (Fig. 1c). These results highlight the extensive involvement of lncRNAs in human tooth development.

Additionally, lncRNAs exhibited lower average expression compared to mRNAs, resulting in fewer highly expressed lncRNAs during human tooth development (Fig. S1). Approximately 1 000 mRNAs together accounted for 50% of the total mRNA expression, while only 10–100 lncRNAs contributed to 50% of the total lncRNA expression (Fig. 1d). Among these, lncRNAs such as *H19*, *MALAT1*, and *MEG8* showed high expression levels in both DE and DM. Additionally, several uncharacterized lncRNAs, including *ENSG00000282885* and *ENSG00000276232*, also exhibited elevated expression in both tissues, highlighting the need for further investigation into their functions (Fig. 1e, f).

Given that lncRNAs are present in similar numbers to mRNAs, their functional roles are of great interest. Studies have suggested that certain mammalian lncRNAs can regulate neighboring developmental regulators in *cis*.^27–29^ During the lineage differentiation of pluripotent cells, lncRNAs transcribed opposite to nearby protein-coding genes regulate the transcription of adjacent genes.^27^ Additionally, the transcription of lncRNAs is often coordinated with the expression changes of neighboring protein-coding genes during embryonic stem cell differentiation.^6^ This coordinated regulation of lncRNA/mRNA gene pairs may be a general feature of cellular differentiation. Thus, *cis*-regulation analysis was conducted to explore the potential functions of lncRNAs based on their proximity to co-located protein-coding genes within 10 kb. Previous studies have emphasized the critical involvement of WNT, FGF, HH, TGF-β, and BMP signaling pathways in tooth development.^1–3,30^ Through *cis*-regulation analysis, we identified 51 key significant signaling molecules associated with tooth development, along with 73 neighboring lncRNAs, suggesting that these lncRNAs may regulate pivotal signaling pathways essential for odontogenesis (Fig. S2a). Additionally, 12 genes linked to human tooth agenesis were identified, along with 18 neighboring lncRNAs (Fig. S2b). Notably, *PANCR*, a lncRNA located near the *PITX2* gene, has been reported to be closely associated with Axenfeld-Rieger syndrome and tooth malformations.^31,32^

### Dynamic expression patterns and functional insights of lncRNAs and mRNAs in the DE

**Fig. 2:**
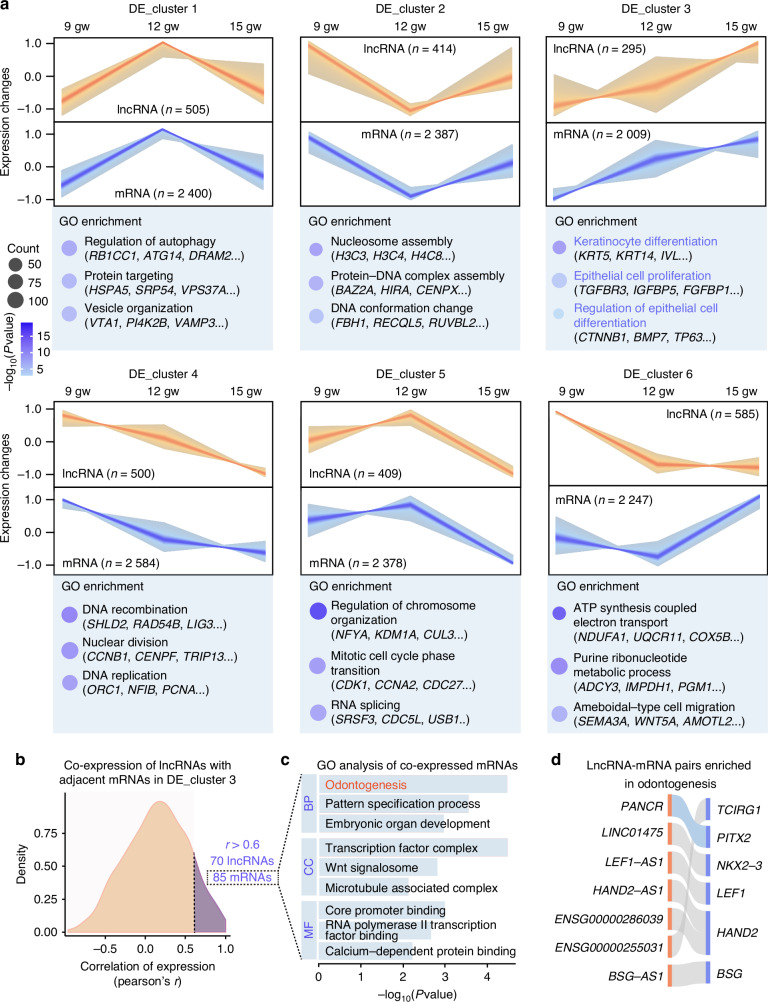
Dynamic expression patterns and functional insights of lncRNAs and mRNAs in DE. **a** Fuzzy c-means clustering identified six distinct temporal expression patterns of lncRNAs and mRNAs in DE. GO enrichment analysis and representative genes were identified for each mRNA cluster. **b** Density distributions of the Pearson correlation coefficients between mRNAs and their adjacent lncRNAs in DE_cluster 3. This analysis revealed a positive correlation (*r* > 0.6) between 85 mRNAs and 70 lncRNAs. **c** GO enrichment analysis of the 85 co-expressed mRNAs shown in (**b**). **d** Alluvial plot depicting lncRNA-mRNA pairs enriched in odontogenesis, as shown in (**c**)

The DE is crucial for tooth development, with the ability to initiate tooth formation at early stages. However, there is limited information on the expression and function of lncRNAs and mRNAs in the DE. As shown in the previous section, numerous lncRNAs and mRNAs were expressed in the DE across all developmental stages. To investigate the dynamic expression patterns of these genes, Fuzzy c-means clustering analysis^33^ was performed, excluding genes with low expression levels where the fragments per kilobase of transcript sequence per million fragments (FPKM) is less than 1 across all samples. This analysis identified six distinct temporal expression patterns of lncRNAs and mRNAs in the DE (Fig. 2a, Tables S1 and S2). Notably, except for DE_cluster 6, lncRNAs in other clusters exhibited expression trends similar to those of the mRNAs. Gene ontology (GO) enrichment analysis was conducted for each mRNA cluster, along with the identification of representative genes. Interestingly, genes in DE_cluster 3 showed a gradual increase in expression over developmental time. These mRNAs were significantly enriched in GO terms related to keratinocyte differentiation, epithelial cell proliferation, and regulation of epithelial cell differentiation (Fig. 2a).

Previous studies have highlighted the co-expression of developmental regulators and their adjacent lncRNAs during mammalian organogenesis.^28^ To further investigate the functions of the lncRNAs in DE_cluster 3, Pearson correlation coefficients (*r*) were calculated between lncRNAs and their adjacent mRNAs across DE samples. This analysis identified 70 lncRNAs that were positively correlated (*r* > 0.6) with 85 mRNAs (Fig. 2b, Table S3). These co-expressed mRNAs were significantly enriched in developmental biological processes, including odontogenesis, pattern specification processes, and embryonic organ development (Fig. 2c). Among these, 7 lncRNA-mRNA pairs were specifically enriched in odontogenesis (Fig. 2d). Notably, *PANCR* exhibited a gradual increase in expression in the DE and was co-expressed with *PITX2*, further supporting its role in tooth development.

### Dynamic expression patterns and functional insights of lncRNAs and mRNAs in the DM

**Fig. 3:**
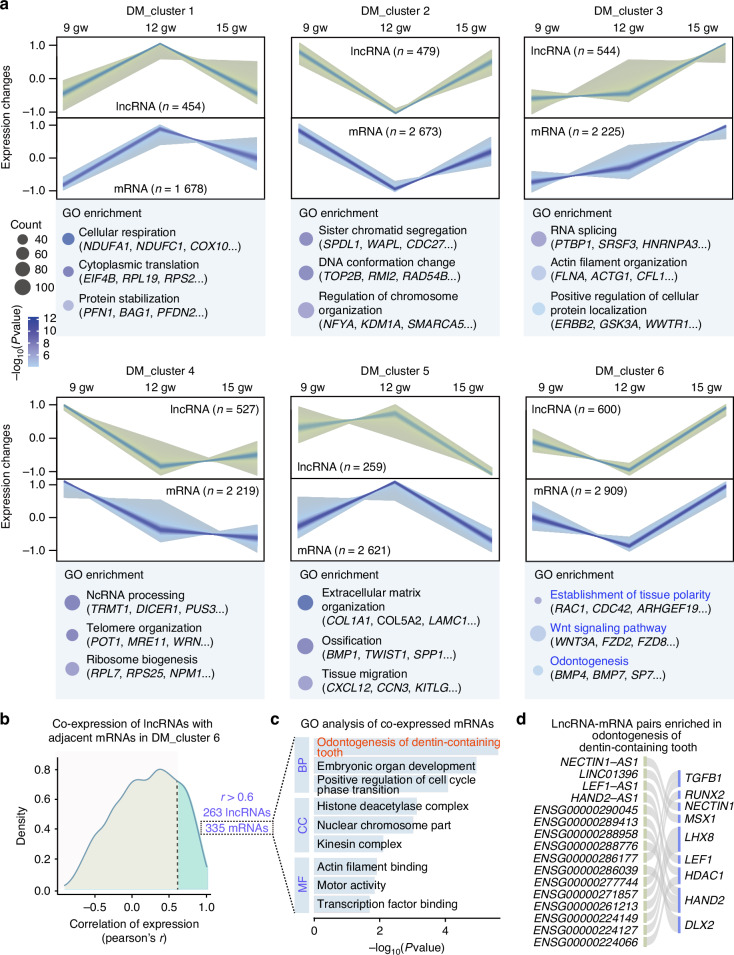
Dynamic expression patterns and functional insights of lncRNAs and mRNAs in DM. **a** Fuzzy c-means clustering identified six distinct temporal expression patterns of lncRNAs and mRNAs in DM. GO enrichment analysis and representative genes were identified for each mRNA cluster. **b** Density distributions of the Pearson correlation coefficients between mRNAs and their adjacent lncRNAs in DM_cluster 6. This analysis revealed a positive correlation (*r* > 0.6) between 335 mRNAs and 263 lncRNAs. **c** GO enrichment analysis of the 335 co-expressed mRNAs shown in (**b**). **d** Alluvial plot depicting lncRNA-mRNA pairs enriched in odontogenesis of dentin-containing tooth, as shown in (**c**)

Next, we proceeded with Fuzzy c-means clustering to investigate the dynamic expression patterns of lncRNAs and mRNAs in the DM. This analysis identified 6 unique temporal patterns of expression in the DM, with lncRNAs and mRNAs in each cluster showing parallel trends (Fig. 3a, Tables S4 and S5). Notably, GO enrichment results in the DM differed from those observed in the DE. In DM_cluster 3, which includes genes with gradually increasing expression, the associated mRNAs were significantly enriched in RNA splicing, actin filament organization, and positive regulation of cellular protein localization. DM_cluster 6, characterized by downregulation at 12 gestation weeks (gw) and peak expression at 15 gw, was enriched in terms such as establishment of tissue polarity, Wnt signaling pathway, and odontogenesis. These findings suggest distinct dynamic expression patterns of odontogenesis-related genes between the DE and DM.

To further investigate the functions of lncRNAs in DM_cluster 6, Pearson correlation coefficients (*r*) were calculated between lncRNAs and their adjacent mRNAs, revealing 263 lncRNAs positively correlated (*r* > 0.6) with 335 mRNAs (Fig. 3b, Table S6). Interestingly, these co-expressed mRNAs were significantly enriched in processes such as odontogenesis of dentin-containing teeth, embryonic organ development, and positive regulation of cell cycle phase transition (Fig. 3c). Among these, 16 lncRNA-mRNA pairs were specifically enriched in odontogenesis of dentin-containing teeth (Fig. 3d).

### Integration of sci-RNA-seq and bulk RNA-seq reveals cell-specific lncRNAs in human tooth germs

LncRNAs are emerging as potential diagnostic markers and therapeutic targets due to their tissue-specific expression.^24,34,35^ Advances in sci-RNA-seq have identified lncRNAs as key markers in specific developmental stages and cell subpopulations across various tissues.^36,37^ To explore the tissue-specific expression of lncRNAs during human tooth development, sci-RNA-seq datasets of developing fetal teeth at the cap stage (9–11 and 12–13 gw) and the early bell stage (14–16 gw), obtained from public databases,^38^ were integrated with previous bulk RNA-seq datasets.

**Fig. 4:**
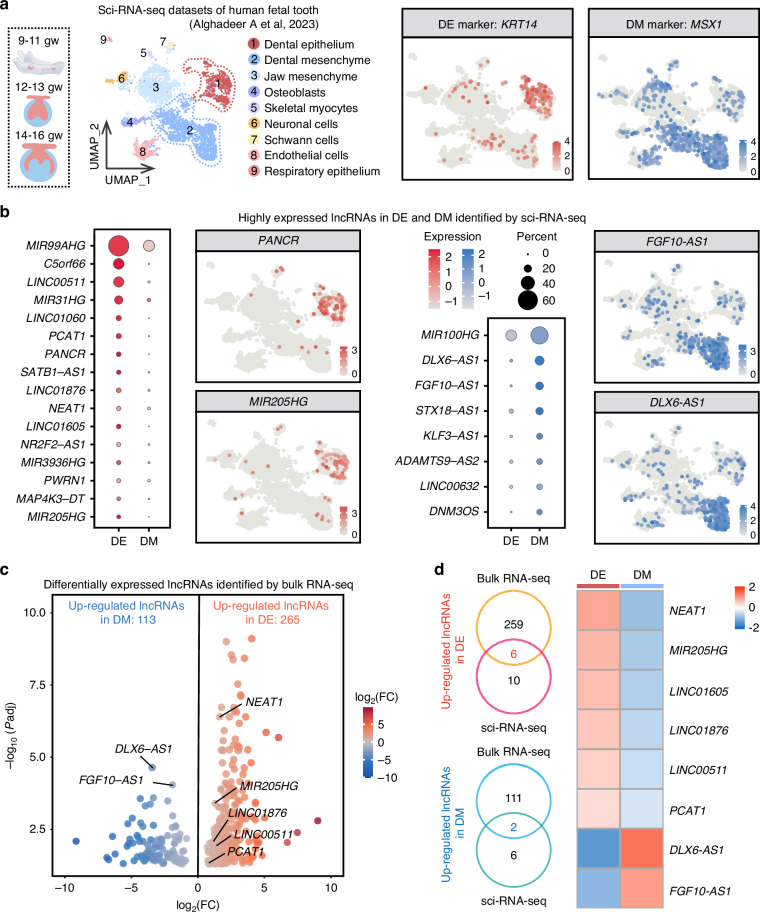
Integration of sci-RNA-seq and bulk RNA-seq reveals cell-specific lncRNAs in developing fetal teeth. **a** UMAP visualization of nine clusters integrated from previously published sci-RNA-seq datasets of developing fetal teeth at the cap stage (9–11 and 12–13 gw) and early bell stage (14–16 gw).^38^ DE and DM clusters annotated with *KRT14* and *MSX1*. **b** Dot plots of highly expressed lncRNAs in DE (red) and DM (blue) from sci-RNA-seq. Feature plot highlighting high expression of *PANCR* and *MIR205HG* in DE, and *FGF10-AS1* and *DLX6-AS1* in DM. **c** Volcano plot of differentially expressed lncRNAs in DE and DM identified by bulk RNA-seq. **d** Venn diagrams and heatmap of up-regulated lncRNAs in DE and DM showing overlap between bulk RNA-seq and sci-RNA-seq

The DE and DM clusters were annotated with *KRT14* and *MSX1* (Figs. 4a and S4a). Cell-specific lncRNAs were identified by screening lncRNAs from the marker genes of these clusters. This analysis revealed 16 lncRNAs that are highly expressed in the DE and 8 lncRNAs that are highly expressed in the DM (Fig. 4b). The feature plot highlighted the high expression of *PANCR* and *MIR205HG* in the DE, while *FGF10-AS1* and *DLX6-AS1* showed high expression in the DM. *PANCR* was confirmed to be specifically expressed in DE, while *DNM3OS* was shown to be specifically expressed in DM in fetal tooth samples at 15 gw using previously published spatial transcriptomic datasets (Fig. S5).^39^ Next, differential expression analysis of lncRNAs was performed using bulk RNA-seq datasets, identifying 265 lncRNAs with significantly higher expression in the DE and 113 lncRNAs with higher expression in the DM (Fig. 4c, Table S7). The mRNAs adjacent to up-regulated lncRNAs in DE were significantly involved in the maintenance of epithelial cell polarity, cell adhesion, and cytoskeleton organization (Fig. S3a). Meanwhile, the mRNAs adjacent to up-regulated lncRNAs in DM were significantly related to filament sliding, oxygen transport, and nucleoside catabolic process (Fig. S3b). By integrating the highly expressed lncRNAs from both bulk RNA-seq and sci-RNA-seq datasets, we identified six lncRNAs specifically highly expressed in the DE and two lncRNAs specifically highly expressed in the DM (Fig. 4d). Additionally, violin plots showed specific high expression of *LINC00511* in the DE and *DLX6-AS1* in the DM, based on sci-RNA-seq data (Fig. S6).

**Fig. 5:**
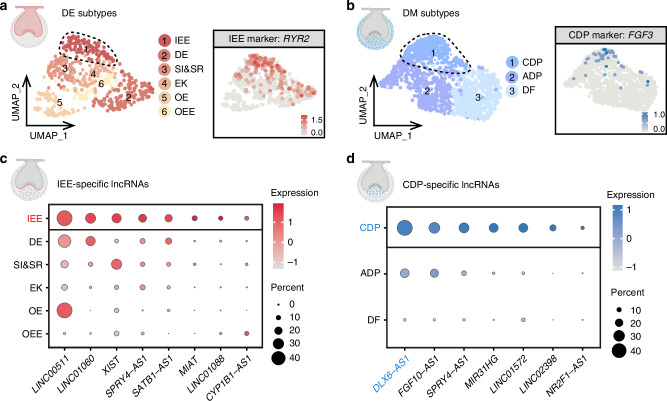
IEE-specific and CDP-specific lncRNAs identified by sci-RNA-seq. **a** UMAP plot of six single-cell clusters in DE from sci-RNA-seq datasets of developing fetal teeth at 9–16 gw. IEE cluster annotated with *RYR2*. IEE inner enamel epithelium, DE dental epithelium, SI&SR stratum intermedium and stellate reticulum, EK enamel knot, OE oral epithelium, OEE outer enamel epithelium. **b** UMAP representation of three single-cell clusters in DM from sci-RNA-seq datasets of developing fetal teeth at 9–16 gw. CDP cluster annotated with *FGF3*. CDP coronal dental papilla, ADP apical dental papilla, DF dental follicle. Dot plots showing IEE-specific lncRNAs (**c**) and CDP-specific lncRNAs (**d**)

Given the cellular heterogeneity of the DE and DM, subcluster analysis was performed on these clusters. The IEE and CDP subclusters are essential for enamel and dentin formation, respectively, and play key roles in tooth regeneration. By analyzing sci-RNA-seq datasets, the IEE (Figs. 5a and S4b) and CDP (Figs. 5b and S4c) subclusters were successfully identified, uncovering 8 IEE-specific lncRNAs (Fig. 5c) and 7 CDP-specific lncRNAs (Fig. 5d). The IEE-specific expression of *LINC00511* and the CDP-specific expression of *NR2F1-AS1* were validated using spatial transcriptomics (Fig. S5). These findings suggest that these lncRNAs could serve as cell markers for the IEE and CDP, potentially involved in enamel and dentin formation.

### CDP-specific lncRNA *DLX6-AS1* enhances odontoblastic differentiation of human primary tooth germ mesenchymal cells (hTGMCs) and human dental pulp stem cells (hDPSCs)

**Fig. 6:**
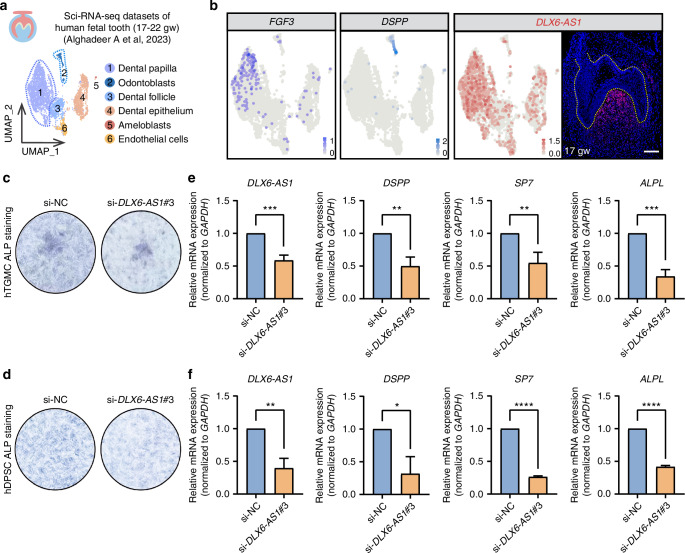
CDP-specific *DLX6-AS1* enhances odontoblastic differentiation of hTGMCs and hDPSCs. **a** UMAP plot of six clusters from previously published sci-RNA-seq datasets of developing fetal teeth at the late bell stage (17–19 and 20–22 gw).^38^ **b** UMAP plot of DP marker *FGF3*, odontoblast marker *DSPP*, and lncRNA *DLX6-AS1*. RNAscope ISH staining of *DLX6-AS1* in human fetal molars at 17 gw. Scale bar, 100 μm. ALP staining after 7-day odontogenic induction in hTGMCs (**c**) and hDPSCs (**d**) with *DLX6-AS1* knockdown. Relative expression of *DLX6-AS1*, *DSPP*, *SP7*, and *ALPL* after 7-day odontogenic induction in hTGMCs (**e**) and hDPSCs (**f**) with *DLX6-AS1* knockdown was measured by qRT-PCR

CDP-specific lncRNA *DLX6-AS1* has been shown to promote odonto/osteogenic differentiation of hDPSCs upon *BMP9* overexpression.^40^ To further investigate its role in dentin formation, sci-RNA-seq datasets from human fetal teeth at the late bell stage (17–20 and 20–22 gw) were analyzed (Figs. 6a and S4d).^38^ Cluster 1, which has been defined as the DP cluster based on high *FGF3* expression, maintains *DLX6-AS1* expression in DP during the late bell stage (Fig. 6b). Additionally, *DLX6-AS1* expression was observed in a subset of cells within cluster 2, identified as the odontoblasts due to high *DSPP* expression (Fig. 6b). RNAscope in situ staining of *DLX6-AS1* in 17 gw fetal teeth confirmed its specific expression in DP, with particularly strong expression in the coronal region (Figs. 6b and S7a). These findings suggest that *DLX6-AS1* plays a role in the process of dentin formation.

The expression of *DLX6-AS1* was found to be higher in the DM at 15 gw compared to 9 and 12 gw based on bulk RNA-seq datasets (Fig. S7b). To further investigate the role of *DLX6-AS1* in odontoblastic differentiation, hTGMCs from 15 gw samples were isolated and cultured in vitro. These cells remain in an undifferentiated state with strong potential for odontoblastic differentiation. Additionally, the expression of *DLX6-AS1* was detected in these cultured cells (Fig. S7c). Three siRNAs were designed to target *DLX6-AS1*, with si-*DLX6-AS1*#3 showing significant knockdown efficiency (Fig. S7d). Knocking down *DLX6-AS1* in hTGMCs resulted in reduced capacity for odontoblastic differentiation, as seen in alkaline phosphatase (ALP) staining (Fig. 6c). Moreover, the expression of odontoblastic genes, such as *DSPP*, *SP7*, and *ALPL*, also decreased significantly after *DLX6-AS1* downregulation (Fig. 6e).

Human DPSCs are considered a rich source of cells for dentin regeneration due to their multi-lineage differentiation potential and easy accessibility from discarded teeth.^41–43^ In this study, we found *DLX6-AS1* expression in hDPSCs (Fig. S7c) and showed that it enhanced odontoblastic differentiation of hDPSCs (Fig. 6d, f). Overall, these results suggest that *DLX6-AS1* could be targeted for promoting dentin formation and aiding in dentin regeneration and repair.

## Discussion

LncRNAs have been identified to play a significant role in tooth development through bulk RNA-seq. Previous studies analyzing rat tooth germs revealed that lncRNAs regulate enamel formation, tooth mineralization, and extracellular matrix remodeling from the bell stage to the secretory stage.^44^ RNA sequencing of miniature pig deciduous tooth germs identified lncRNAs participating in the development from the cap stage to the bell stage, co-expressing with extracellular matrix receptor-associated genes.^17^ However, the majority of these studies have centered on animal dental tissues, posing a challenge in applying the results to human tooth development and regeneration. Furthermore, the average expression levels of lncRNAs in the entire tooth germs fail to capture tissue-specific lncRNA expression patterns.^45–47^

Tooth development is a biological process that is driven by the interaction between the DE and DM. Both of these tissues exhibit significant heterogeneity, with distinct gene expression patterns. However, studying lncRNAs in human DE has been challenging due to their degeneration post-eruption, limited primary cell sources, and difficulties maintaining primary cells.^48^ Tissue recombination studies have demonstrated that the human DE at the cap stage retains tooth-inductive capability, containing signaling molecules that induce mesenchymal differentiation.^49,50^ In this study, we conducted bulk RNA-seq analysis on isolated human DE and DM tissues to comprehensively investigate the expression of lncRNAs during human tooth development. Dynamic lncRNAs were found to be co-expressed with neighboring protein-coding genes, a pattern consistently observed in organ development.^6,27^ The coordinated regulation of lncRNA/mRNA pairs may represent a general feature of odontogenesis.

Recent advancements in sci-RNA-seq have enabled the analysis of gene expression during organ development at the single-cell level. These technologies have identified cell-type-specific lncRNAs that exhibit strong correlation with known cell-type marker genes.^36,37^ By integrating human tooth sci-RNA-seq and bulk RNA-seq datasets, we identified lncRNAs that are highly expressed in DE and DM. For instance, the nuclear paraspeckle assembly transcript 1 (*NEAT1*) is highly expressed in DE. *NEAT1*, which is widely expressed in mammalian cells, plays pivotal roles in various biological and pathological processes by recruiting or sequestering RNA-/DNA-binding proteins to regulate gene transcription, splicing, RNA stability, or translation.^51^ Previous studies have shown that *NEAT1* modulates the alternative splicing of the microphthalmia-associated transcription factor, influencing the proliferation of retinal pigment epithelium.^52^

Through subcluster analysis, we also identified lncRNA markers for IEE and CDP through sci-RNA-seq. The X-inactive specific transcript (*XIST*) gene, a master regulator of X chromosome inactivation in mammals, is highly expressed in IEE. Notably, a case report of severe X-linked hypohidrotic ectodermal dysplasia with tooth agenesis highlighted the loss of *XIST* on the X chromosome, leading to disruption of the *EDA* gene.^53^ Furthermore, we demonstrated that the CDP-specific *DLX6-AS1* enhances odontoblastic differentiation of hTGMCs and hDPSCs. *DLX6-AS1* has also been shown to promote odonto/osteogenic differentiation of human dental pulp cells upon *BMP9* overexpression.^40^ Additionally, mouse *Dlx6os1*, the homolog of the human *DLX6-AS1* gene, was identified as a signature gene in the CDP of the mouse molar at embryonic day 16.5.^54^ Although most lncRNAs show species specificity due to their rapid evolution and low sequence conservation, some lncRNAs remain conserved across species based on sequence similarities.^28^ Homologous lncRNAs in conserved animal models can provide insights into tooth development mechanisms, establish reliable animal models, elucidate molecular networks, and address the challenge of obtaining human embryos for dental research. In the future, constructing experimental mouse models based on *Dlx6os1* will help further investigate its role and regulatory mechanisms in dentin development and regeneration.

In addition to cell-type specificity, lncRNAs exhibit stage-specific expression patterns during development.^28,36,37^ Their dynamic expression is often coordinated with the expression of neighboring genes and is closely associated with tissue differentiation.^36^ Tooth development is a complex and dynamic process, with distinct gene expression profiles observed in DE and DM at different stages.^2,3,30^ In this study, we analyzed the mRNA and lncRNA expression profiles during the late bud, cap, and early bell stages of DE and DM. We found that both mRNAs and lncRNAs exhibit similar dynamic expression patterns. These lncRNAs are co-expressed with adjacent mRNAs and play a crucial role in odontogenesis. Additionally, dynamic lncRNAs in DE and DM are predicted to be involved in epigenetic regulatory processes, such as transcription factor binding and core promoter binding. LncRNAs have been reported to play essential roles in embryonic development, organogenesis, and tissue homeostasis at both the transcriptional and post-transcriptional levels.^5,55^ Studies based on in vitro odontogenic differentiation systems can provide valuable insights into the molecular mechanisms of lncRNA function within the regulatory networks of dental mesenchymal stem cells through epigenetic regulation.^56–60^ However, the epigenetic roles of lncRNAs in lineage determination and cellular differentiation during tooth development require further in vivo investigation.

This study not only deepens understanding of lncRNAs in human tooth development but also identifies those adjacent to genes associated with tooth agenesis. Recent research has shown that the lncRNA *CHASERR* regulates the expression of the *CHD2* gene in *cis*, and a single-copy deletion of *CHASERR* leads to increased *CHD2* protein levels, which results in neurodevelopmental defects.^61^ This finding indicates that lncRNAs are involved in a broader range of human diseases, particularly those located upstream of genes associated with monogenic disorders. In this study, we identified *PANCR*, a lncRNA located near the *PITX2* gene, which has been reported to be closely associated with Axenfeld-Rieger syndrome.^31,32^ Notably, *PITX2* is a crucial transcription factor in tooth development, and mutations in *PITX2* are known to cause tooth and craniofacial agenesis.^62,63^ A familial case of Axenfeld-Rieger syndrome, characterized by clinical manifestations including tooth malformations, revealed a significant deletion that encompassed both *PITX2* and *PANCR*.^32^ Interestingly, no mutations were found in the coding regions or exon-intron junctions of *PITX2*, suggesting that *PANCR* may play a role in modulating tooth development through its interaction with *PITX2*. Moreover, we found that *PANCR* is specifically expressed in DE and co-expressed with *PITX2*. The expression of *PANC*R in DE progressively increases during development, further supporting its potential role in odontogenesis.

LncRNAs, characterized by their distinct expression patterns and tissue specificity, hold significant promise as biomarkers and potential intervention targets for tissue regeneration and gene therapy. Adeno-associated virus (AAV) vectors carrying lncRNAs serve as effective delivery tools, demonstrating therapeutic potential for clinical applications.^64^ For instance, AAV9-*H19* vectors targeting muscle tissue have been shown to reverse pathological cardiac hypertrophy.^65^ With advancements in regenerative medicine, exosome-derived lncRNAs have emerged as a critical component in drug delivery systems for stem cell-based therapies.^66^ Exosomes derived from bone marrow mesenchymal stem cells can enhance the expression of *H19*, contributing to the treatment of osteogenic differentiation issues and poor fracture healing associated with obesity.^67^ Furthermore, exosomal *MALAT1* has proven effective in preventing osteoporosis in ovariectomized mouse models.^68^ Several lncRNAs, including *H19* and *MALAT1*, have been reported to regulate the odontogenic/osteogenic differentiation of DPSCs in vitro.^14,16,58^ In this study, we observed that lncRNAs such as *H19* and *MALAT1* are highly expressed in both the DE and mesenchyme during the early stages of human tooth development. AAV gene therapy and exosome-mediated delivery, targeting these lncRNAs involved in tooth development, represent promising clinical strategies for future dental regeneration therapies.

With the abundance of sci-RNA-seq and RNA-seq datasets available, there are now more opportunities for data mining and gaining a deeper understanding of lncRNA functions. This research illuminates the extensive landscape of lncRNAs in developing human teeth, paving the way for further studies on lncRNAs in tooth development, regeneration, and tooth agenesis.

### Limitations of the study

Our research focuses on the early phases of tooth development, specifically from the late bud to early bell stages, and does not address the later stages, creating a gap in comprehension of the entire developmental trajectory. Sci-RNA-seq has limitations in studying lncRNA expression, including reduced sensitivity to low-abundance lncRNAs, incomplete coverage, and challenges with annotation. These limitations highlight the importance of future studies utilizing advanced technologies to explore the involvement of lncRNAs, such as *DLX6-AS1*, throughout the entire process of tooth development.

## Materials and methods

### Tissue collection and dissection

Human fetal tissues were dissected in cold RNase-free Phosphate-Buffered Saline (PBS) (Servicebio, Wuhan, China) on ice. The mandibular molar tooth germs were carefully isolated under a dissection microscope using microtools. After washing with PBS, the tooth germs were incubated in 3 mg/mL Dispase II (Sigma-Aldrich, MO, USA) for 5 min at room temperature, followed by neutralization with Dulbecco’s PBS (Beyotime, Shanghai, China). The DE and mesenchymal tissues were then separated using micro forceps and stored separately in RNAlater (Invitrogen, CA, USA) to preserve RNA integrity.

### Bulk RNA-seq

Bulk RNA-seq was conducted by Novogene Company (Beijing, China). Total RNA was extracted using Trizol reagent, and sequencing libraries were prepared with the RNA Library Prep Kit for Illumina according to the manufacturer’s instructions. Qualified libraries were pooled and sequenced on Illumina platforms using a paired-end 150 bp read length strategy. Raw reads were aligned to the human reference genome GRCh38 (hg38). Basic quality statistics of the raw reads were assessed using Fastp (version 0.23.1). Gene expression levels were measured in FPKM.

### GO enrichment analysis

GO enrichment analysis was performed on specific mRNAs using the NovoMagic platform. Alternatively, mRNAs adjacent to specific lncRNAs, as predicted, were also subjected to GO enrichment analysis.

### Fuzzy C-means clustering

LncRNAs and mRNAs from three developmental stages were clustered using the Mfuzz package in R with the fuzzy c-means algorithm.^33^ Genes with low expression (FPKM < 1 across all samples) were excluded from this analysis.

### Correlation between lncRNA and adjacent mRNA expression

Adjacent mRNAs within 10 kb upstream and downstream of the lncRNAs were identified based on the genome annotation file, and the expression levels of target lncRNAs and their adjacent mRNAs across samples were analyzed. The Pearson correlation coefficients (*r*) and significance test results (*P* value) between lncRNA and mRNA expression were calculated using the cor.test function in R. The Pearson correlation coefficient *(r*) measures the degree of linear correlation, with values ranging from −1 to +1. A value of +1 indicates a perfect positive correlation, −1 indicates a perfect negative correlation, and 0 indicates no linear correlation. Plot the density distribution of *r* values using ggplot2 in R. In this study, the criteria of *r* > 0.6 and *P* < 0.05 were used to assess significant positive correlations between the expression of lncRNAs and mRNAs. We refer to these closely associated lncRNA and mRNA genes as lncRNA/mRNA gene pairs.

### Differential expression analysis

Differential expression analysis was performed using the edgeR software. Significance levels were assessed based on *P* values or adjusted *P* values (*p*adj). The *p*adj values are derived by applying multiple testing correction to the *P* values, accounting for high false positive rates. A threshold of *P*adj ≤ 0.05 was used to define significant differential expression.

### Analysis of sci-RNA-seq datasets

Seurat V4 was used to integrate single-cell datasets from human tooth germs at the cap stage (9–11 and 12–13 gw) and the early bell stage (14–16 gw), as well as from the late bell stage (17–19 and 20–22 gw) using the same approach.^38^ Data normalization, principal component analysis (PCA), and UMAP clustering were performed using the SCTransform, RunPCA, and RunUMAP functions. Batch effects between samples were corrected using the RunHarmony function. Marker genes for each cell cluster were identified with the FindAllMarkers function, and cell types were assigned based on these markers. Subcluster analysis was performed to investigate the heterogeneity within the DE and DM populations. Cell-specific lncRNAs were identified by screening lncRNAs from the marker genes of each cell type. The expression of these cell-specific lncRNAs was visualized using FeaturePlot, DotPlot, and VlnPlot functions, generating feature plots, dot plots, and violin plots, respectively.

### Spatial transcriptomics

The spatial expression patterns of tissue-specific lncRNAs were visualized using the SpatialFeaturePlot function from the Seurat package in R, based on the human tooth germ spatial transcriptomics data in our previous study.^39^

### RNAscope in situ hybridization (ISH)

Human fetal craniofacial tissues from 17 gw were analyzed using ISH with the RNAscope Multiplex Fluorescent Reagent Kit v2 (Advanced Cell Diagnostics, CA, USA). Briefly, tissues were fixed in 4% paraformaldehyde overnight at 4 °C before decalcification in 10% EDTA for 2 weeks, and subsequently dehydrated and embedded in paraffin. Sections were cut to a thickness of 6 μm. Target retrieval was performed for 15 min at 95–100 °C, followed by protease treatment for 30 min at 40 °C. The *DLX6-AS1* probe (#569491-C2, Advanced Cell Diagnostics) was then hybridized for 2 h at 40 °C, followed by amplification with RNAscope reagents. Signals were visualized using TSA Plus Cyanine 3. The human positive control probe (#320861, Advanced Cell Diagnostics) was used as the positive control, while PBS without any probe was served as the negative control.

### Cell culture

Primary hTGMCs were prepared from isolated fetal tooth germ mesenchymal tissues at 15 gw. The mesenchymal tissues were incubated in 0.25% Trypsin-EDTA (Gibco, NY, USA) for 5 min at 37 °C. The cells were centrifuged and expanded with Dulbecco’s modified Eagle’s medium (DMEM; Biosharp, Hefei, China) supplemented with 10% fetal bovine serum (FBS), 100 units/mL penicillin, and 100 mg/mL streptomycin, and incubated at 37 °C in 5% CO_2_. The hDPSCs were cultured under good manufacturing practice conditions as described previously.^69^ Cultured hTGMCs and hDPSCs at passage three were used for siRNA transfection and odontoblastic differentiation experiments. The media was changed every 2 days.

### Transfection of siRNAs and odontoblastic differentiation

Cells were transfected with 40 nmol/L siRNAs to knock down *DLX6-AS1* expression. Three siRNAs, synthesized by Sangon Biotech (Shanghai, China), were diluted in Opti-Eagle’s minimal essential medium (Invitrogen) and transfected using Lipofectamine RNAiMAX (Invitrogen), with a nontargeted siRNA as the negative control. The siRNA sequences are provided in Table S8. After 12 h, the medium was replaced with osteogenic induction differentiation medium (Procell, Wuhan, China). Following 7 days of induction, cells were subjected to ALP staining and RNA extraction.

### ALP staining

ALP staining was performed using the Pluripotent Stem Cell Alkaline Phosphatase Color Development Kit (Beyotime) following the manufacturer’s instructions. Briefly, cells were fixed in 4% paraformaldehyde, washed with PBS, and stained with ALP stain working solution to assess odontoblastic differentiation. The intensity of ALP staining was then visually evaluated to determine the differentiation status.

### RNA extraction and qRT-PCR

RNA was extracted from the collected samples using the *SteadyPure* Quick RNA Extraction Kit (AG, Hunan, China) and analyzed by qRT-PCR. The RNA was reverse transcribed into cDNA using an oligo (dT) primer and Reverse Transcriptase (Tiangen, Beijing, China). qRT-PCR was performed with a SYBR qPCR SuperMix Plus kit (Novoprotein, Suzhou, China) on a CFX96 Touch Real-time PCR Detection System. *GAPDH* was used as an internal control to normalize the target genes. Fold changes in expression were calculated using the relative Ct method. The primer sequences are listed in Table S9.

### Statistical analysis

Bioinformatics and statistical analyses were performed using R (version 4.2.2), the NovoMagic platform, and GraphPad Prism (version 8.0). Statistical comparisons were conducted using the Student’s *t*-test or one- or two-way ANOVA. Data, presented as the mean ± SD, were derived from at least three independent experiments. *P < 0.05, **P < 0.01, ***P < 0.001, ****P < 0.000 1 were considered significant, and ns was not significant. All sample experiments were repeated three times, independently.

## Supplementary information

Supplementary Figures

Supplementary Tables

## Data Availability

The raw sequence data reported in this paper have been deposited in the Genome Sequence Archive^70^ in the National Genomics Data Center,^71^ China National Center for Bioinformation/Beijing Institute of Genomics, Chinese Academy of Sciences (GSA-Human: HRA010578) that are publicly accessible at https://ngdc.cncb.ac.cn/gsa-human. All other relevant data supporting the key findings of this study are available within the article and its Supplementary Information files or from the corresponding author upon reasonable request.
