# Supplementary material for: Profiling and functional characterization of long noncoding RNAs during human tooth development
Source: Int J Oral Sci. 2025 May 9;17:38. doi: 10.1038/s41368-025-00375-7 (PMC12064826; doi:10.1038/s41368-025-00375-7)
Supplement: Supplementary file 1 — Supplementary Figures [file 41368_2025_375_MOESM1_ESM.doc]

**Supplementary Figures**

**Supplementary Figure 1**

**
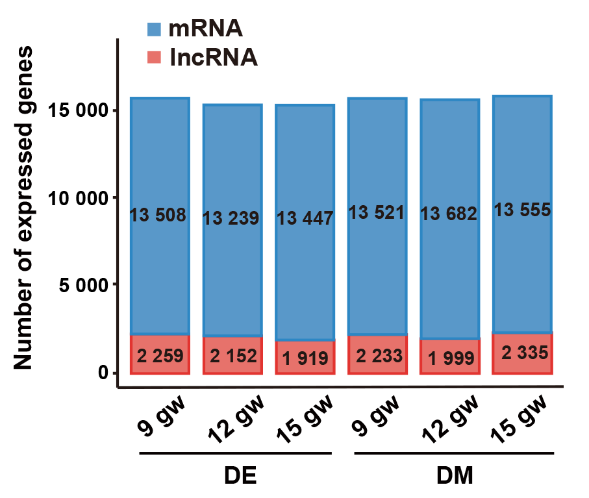
**

**Fig. S1** The number of genes expressed in the DE and DM across the three developmental stages, excluding those with FPKM < 1 in all samples.

**Supplementary Figure 2**

**
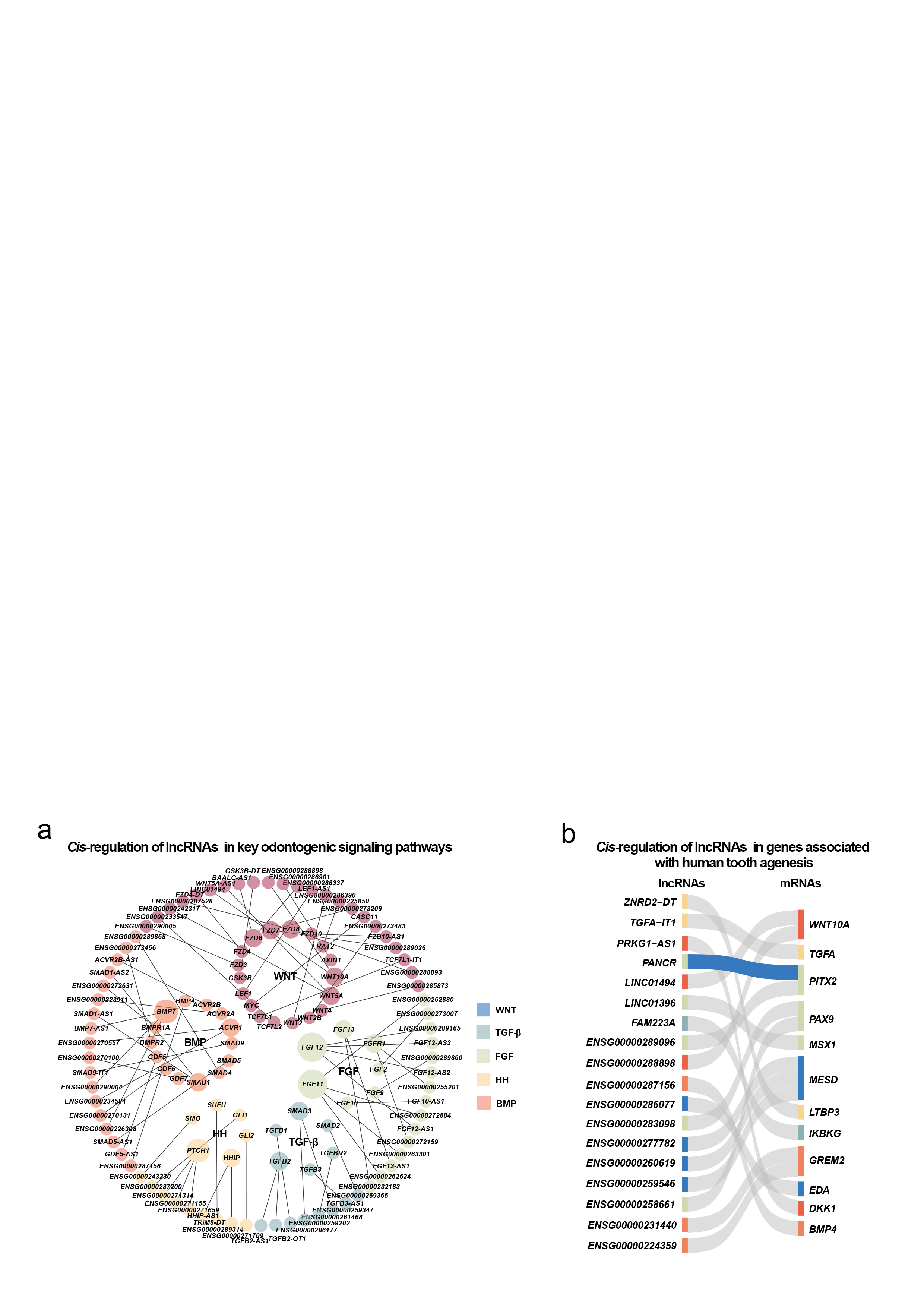
**

**Fig. S2** *Cis*-regulatory relationship diagrams of signaling molecules involved in tooth development (**a**) and genes related to human tooth agenesis (**b**), along with their adjacent lncRNAs within 10 kb.

**Supplementary Figure 3**


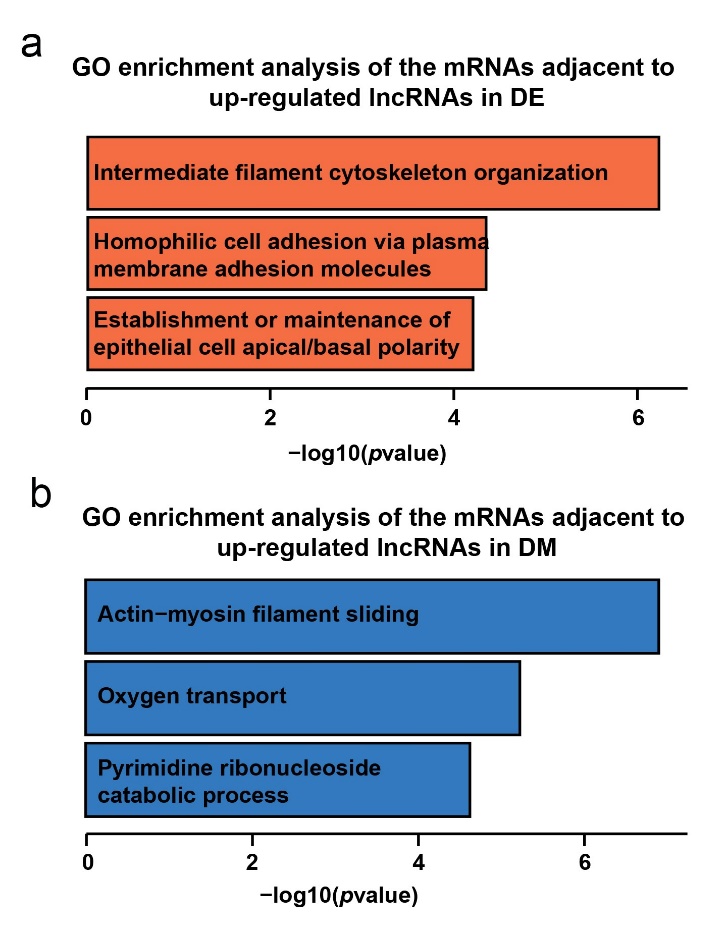


**Fig. S3** GO enrichment analysis of the mRNAs adjacent to up-regulated lncRNAs in DE (a) and DM (b) identified by bulk RNA-seq.

**Supplementary Figure 4**

**
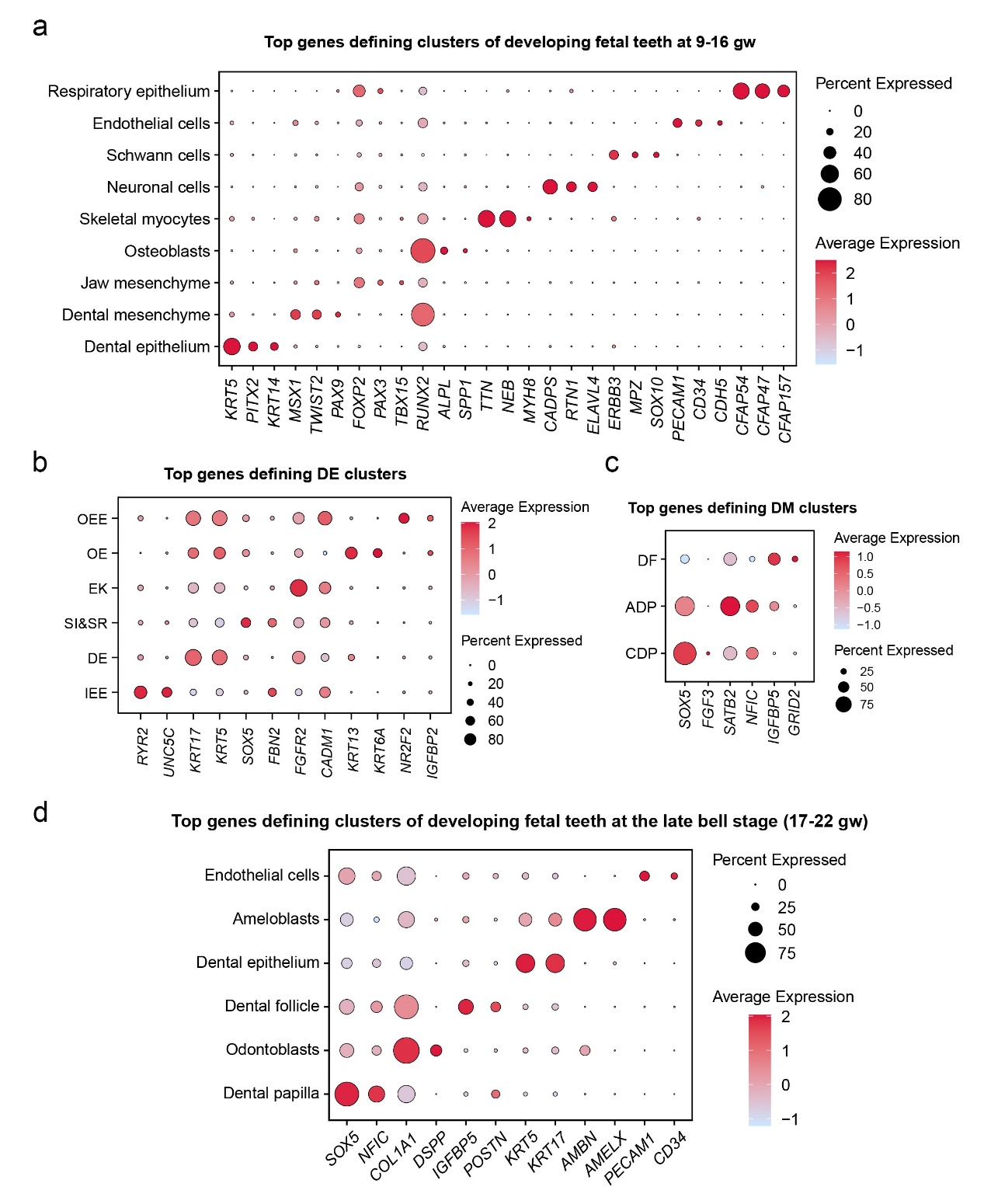
**

**Fig. S4** Top genes defining clusters of developing fetal teeth identified by sci-RNA-seq.

**a** Top genes defining clusters of developing fetal teeth at the cap stage (9-11 and 12-13 gw) and early bell stage (14-16 gw). **b** Top genes defining DE clusters at 9-16 gw. **c** Top genes defining DM clusters at 9-16 gw. **d** Top genes defining clusters of developing fetal teeth at the late bell stage (17-22 gw).

**Supplementary Figure 5**


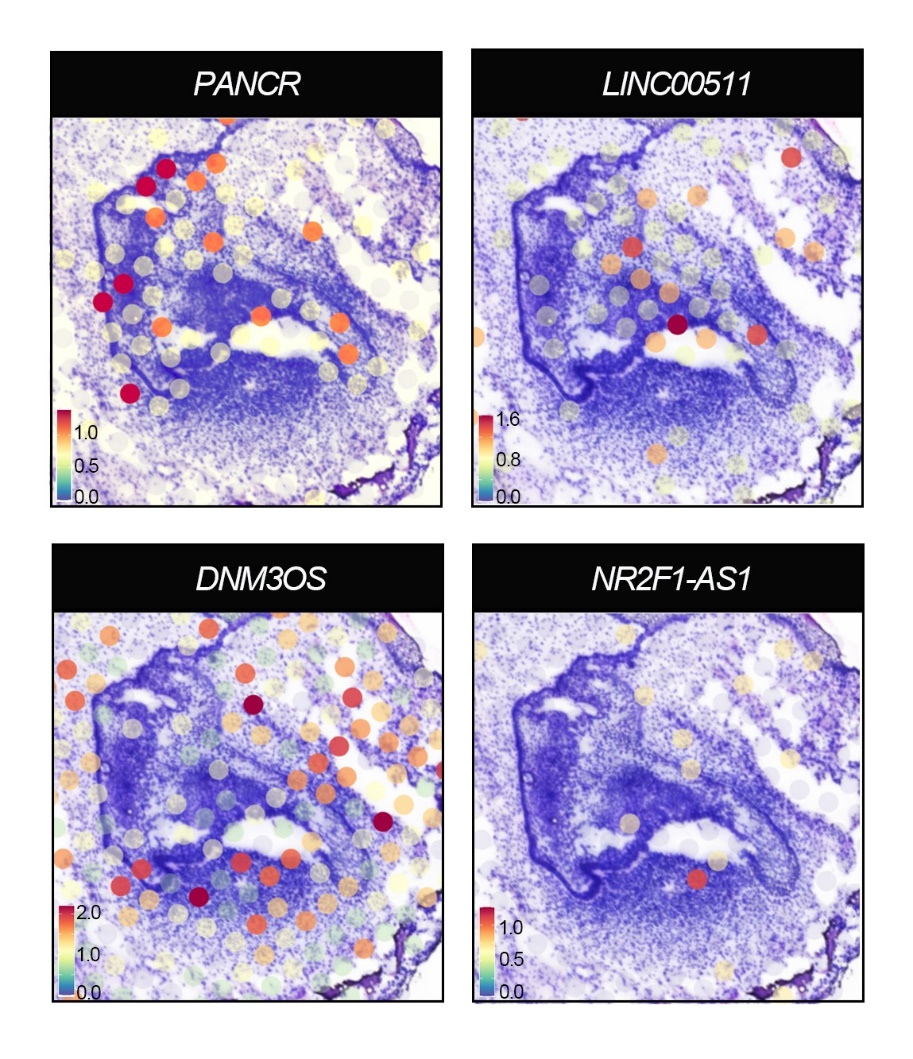


**Fig. S5** Spatial expression of tissue-specific lncRNAs in the fetal tooth sample at 15 gw, analyzed using spatial transcriptomic datasets.

**Supplementary Figure 6**


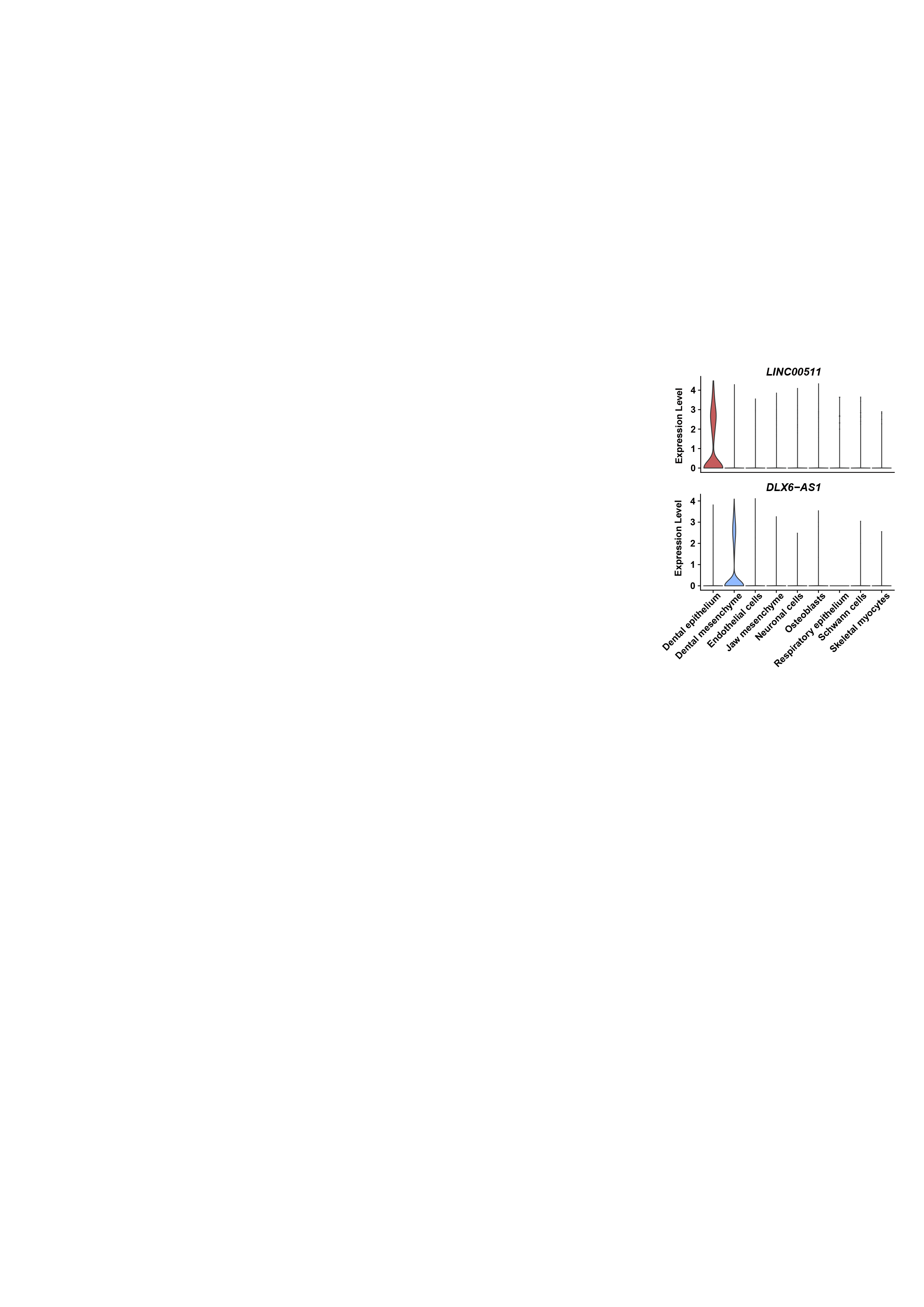


**Fig. S6** Violin plots showing specific high expression of *LINC00511* in DE and *DLX6-AS1* in DM, based on sci-RNA-seq datasets of developing fetal teeth at 9-16 gw.

**Supplementary Figure 7**


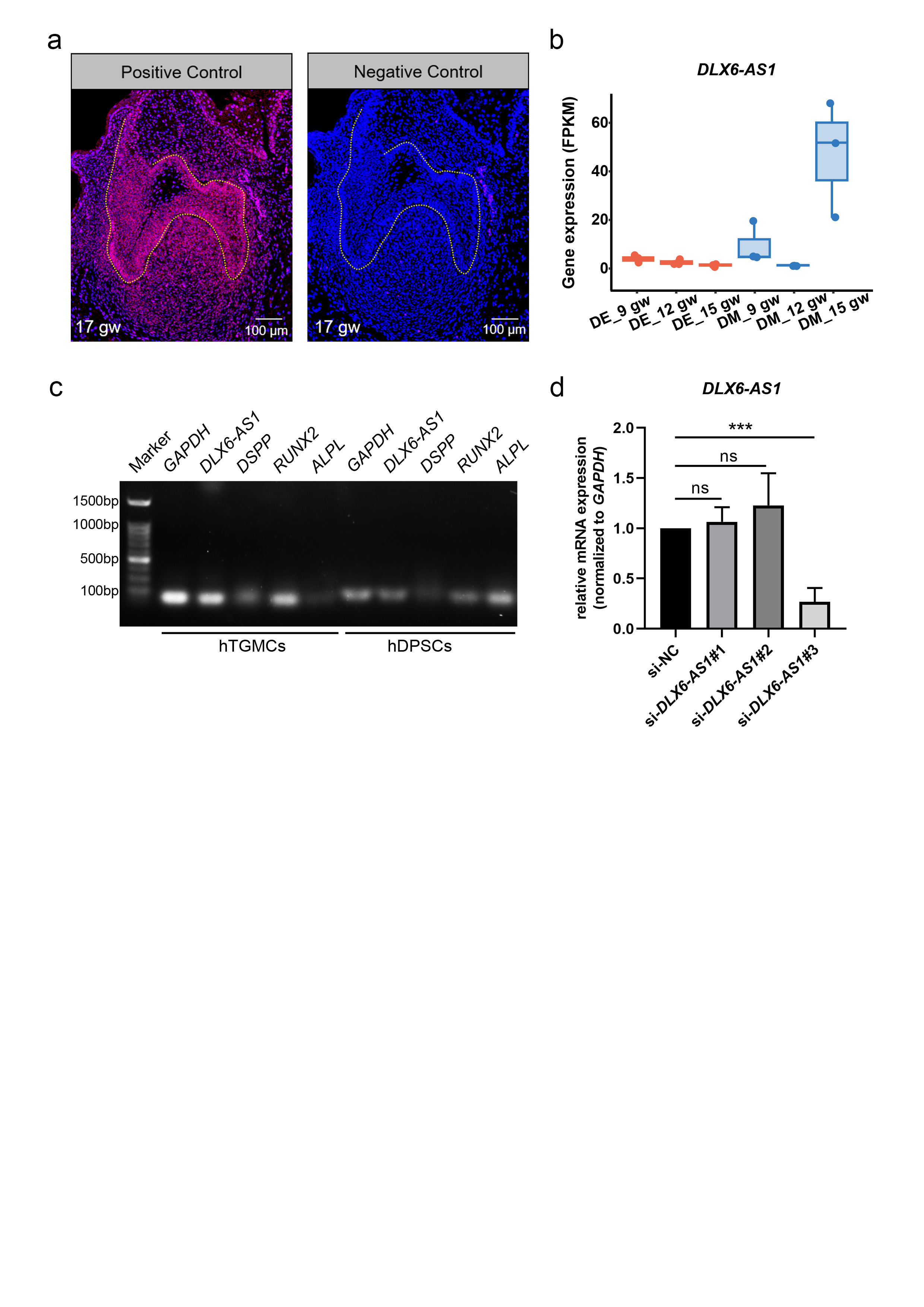


**Fig. S7** The expression levels of *DLX6-AS1* in dental mesenchymal tissue, hTGMSc, and hDPSCs.

**a** Positive and negative controls in 17 gw fetal teeth confirmed by RNAscope *in situ* hybridization. **b** Boxplots showing the expression (FPKM) of *DLX6-AS1* in DE and DM across the three developmental stages, through bulk RNA-seq. **c** The expression of *DLX6-AS1*, *DSPP*, *RUNX2*, and *ALPL* was detected in hTGMCs and hDPSCs by qRT-PCR and agarose gel electrophoresis. **d** Relative expression of *DLX6-AS1* after siRNA transfection in hTGMCs was measured by qRT-PCR.
